# Knowledge, attitude, and practice of asthma among the adults in Shendi locality, Sudan: A cross-sectional study

**DOI:** 10.1097/MD.0000000000040395

**Published:** 2024-11-01

**Authors:** Sara Elawad, Sabaa I. Saad-Omer, Shaima Omer Mohamed Elawad, Mohamed H. Elbadawi, Sahar A.A. Ahmed, Sara B. Bashir, Selma A.H. Abdelmutaleb, Sarah O. Mohamed, Suad B. Babiker, Sara M.S. Osman, Mohamed A.H. Fadul, Esameldeen O.M. Abdalkareem, Ahmed Balla M. Ahmed, Ali Galal

**Affiliations:** a University of Khartoum, Faculty of Medicine, Khartoum, Sudan.

**Keywords:** asthma, attitude, KAP asthma, knowledge, practice, rural

## Abstract

Asthma is a chronic inflammatory condition leading to intermittent airway narrowing and obstruction. Although millions suffer from asthma globally, there is limited data from African countries, particularly Sudan. This study addresses the gap in research on asthma knowledge, attitudes, and practices among the adults in Shendi locality, Northern Sudan. This cross-sectional, community-based study was conducted in Kabushiya village, Shendi, Sudan, involving 148 participants selected through multistage random sampling. Data were collected via face-to-face interviews using a validated, structured questionnaire. Statistical analyses included Mann–Whitney, Kruskal–Wallis, Chi-square, Fisher exact tests, and multiple linear regression. Participants demonstrated high levels of knowledge, positive attitudes, and good practices regarding asthma. Knowledge scores were significantly associated with marital status, education, occupation, and income (*P* values: .040, .003, .000, and .006, respectively), with the highest medians among divorced individuals, those with higher education, professional workers, and people earning <50,000 SDGs (medians: 17, 16, 17, and 16, respectively). Attitude scores varied significantly by marital status, education, occupation, and family asthma history (*P* values: .001, .000, .003, and .016, respectively), with highest scores among divorced individuals, those with high school or higher education, professionals, and those with a first-degree relative with asthma (medians: 6, 5, 5, and 5, respectively). Patient practice scores differed by marital status and income (*P* values: .026 and .006, respectively), with higher scores among singles and those earning <50,000 SDGs. Co-patient practice scores were significantly influenced by occupation, personal or familial asthma experience, and family asthma status (*P* values: .026, .003, and .000, respectively). No significant differences were observed in co-patient practice scores between groups. The study highlights a generally good level of asthma knowledge and positive attitudes among participants, with some variability in practices based on socio-demographic factors. These findings emphasize the need for targeted education and intervention programs to improve asthma management in rural Sudan.

## 1. Introduction

Asthma is a common chronic inflammatory airway disease characterized by airway inflammation, which leads to occasional airflow obstruction and bronchial hyper-responsiveness.^[1]^ It is diagnosed based on typical symptoms such as wheezing, shortness of breath, and cough, as well as the measurement of lung function. Spirometry and peak expiratory flow measurement are the primary tests for assessing lung function, while invasive tests like bronchoprovocation should be reserved for cases with atypical presentations.^[2]^

Globally, approximately 300 million people suffer from asthma, making it account for 1% of all diseases. It causes 180,000 deaths and 15 million disability-adjusted life years annually.^[3]^ The prevalence of asthma increases by about 50% every decade.^[4]^ Although data from Africa are limited, evidence suggests that prevalence rates have risen over the past 2 decades, with sub-Saharan Africa reporting rates of 6 to 20%.^[5,6]^

Long-term asthma care includes pharmacotherapy, home treatment, and avoiding triggers. While pharmacotherapy remains the cornerstone of treatment, managing asthma can be significantly improved by avoiding triggers such as cold air, smoke, dust, strong fragrances, and upper respiratory tract infections.^[7]^ Asthma impacts both physical and mental health. The stigma surrounding asthma, particularly in rural areas due to a lack of health awareness and limited access to healthcare facilities, often leads to inadequate asthma control.^[8]^

Each patient and their family should have a plan to handle acute attacks. Studies indicate that less education increases the likelihood of uncontrolled asthma; therefore, managing asthma requires more than just medication.^[9]^

In Sudan, uncontrolled asthma is quite prevalent, affecting 84.5% of patients.^[10]^ Many patients in Khartoum tertiary hospitals continue to experience asthma symptoms despite the availability of effective treatments.^[6,11]^ This situation may be due to factors such as lack of awareness, negative attitudes, or improper behaviors, such as missing follow-up appointments, improper inhaler use, or misconceptions about asthma. A hospital-based study in Khartoum revealed low levels of knowledge, attitudes, and practices among mothers of asthmatic patients.^[12]^ Although successful asthma treatment relies on adequate education, a positive attitude, and proper practices,^[12]^ there are no studies addressing the knowledge, attitudes, and practices regarding asthma in the adult population of rural Sudan. Therefore, this study aimed to assess the knowledge, attitudes, and practices related to asthma among the adults in Kabushiya village, Shandi locality, Sudan, to help develop effective management plans.

## 2. Methods

### 2.1. Study design and setting

This was a cross-sectional community-based study conducted in March 2022. It aimed to investigate the knowledge, attitudes, and practices related to asthma among the adults in Kabushiya village, located in the Shendi locality of the River Nile State in Sudan. Kabushiya is situated east of the Nile River and had a population of 66,000 according to the 2020 census. The village is divided into 12 residential areas. Kabushiya is home to one of Sudan’s first general hospitals and is served by health units in nearby villages such as Deim Al-Qarray, Goz Bara, Al-Hammadab, and Meroe.

### 2.2. Study participants

The study participants included all residents of Kabushiya village who were 18 years or older at the time of data collection in March 2022. During the household visits, all adults present at the time of data collection were invited to participate, ensuring a comprehensive representation of adult individuals in each household. Participants were required to provide informed consent, and individuals who were unable or unwilling to do so were excluded from the study.

Participants were categorized into patients, co-patients, and others. Patients referred to individuals who had been diagnosed with asthma. Co-patients included family members or caregivers in the same household who regularly took care of or interacted with the asthma patients. We included 9 patients and 35 co-patients in the study, representing the role of caregivers in asthma management. The remaining participants were those without a direct role in asthma care.

The sociodemographic, medical information, knowledge, and attitude sections were completed by all participants. The patient practice section was completed by asthma patients, while the co-patient practice section was filled out by individuals identified as co-patients.

### 2.3. Data collection

Data were collected in March 2022 by trained medical students who conducted interviews in the participants’ households to ensure a comfortable environment for the respondents. The data collectors received thorough training on the study’s objectives and the questionnaire to ensure consistency and accuracy in data collection. The interviews were conducted in the afternoons to maximize participant availability. Each adult present at the time of the interview was asked to respond to the questionnaire. The overall response rate for the study was 67%.

### 2.4. Sample size and sampling technique

The sample size was calculated using the formula for descriptive studies^[13]^:


$$\begin{array}{l} n=NZ^{2}P\left(1-P\right)/d^{2}\left(N-1\right)+Z^{2}P\left(1-P\right) \end{array}$$

where:

n = sample size with finite population

correction

N = population size = 25,000

Z = Z statistic for a level of confidence. For 95% confidence = 1.96

P = expected proportion (in proportion of 1) = 10.8%

d = precision (in proportion of 1) = 0.05

The calculated sample size was 147. Since we used multistage random sampling, we multiplied by the design effect, which was set to 1.5.^[14]^ This adjustment accounts for the increased variability due to clustering in multistage sampling. A design effect of 1.5 is commonly used when no detailed intracluster correlation is available, ensuring the sample size remains adequate. Therefore, the adjusted sample size is 221.

A multistage random sampling method was used to select them. First, 3 out of the 12 residential areas in Kabushiya were randomly chosen. Then, within those 3 areas, houses were selected using a systematic random sampling technique for the second stage.

### 2.5. Study tool

Data collection was conducted through face-to-face interviews. The study used a structured questionnaire designed by the authors, which was validated and piloted before use.

The questionnaire consisted of 5 sections with closed-ended questions: 1 for socio-demographic and medical information, and 3 others to assess knowledge, attitudes, and practices. The sociodemographic, medical information, knowledge, and attitude sections were completed by all participants. The patient practice section was completed solely by individuals diagnosed with asthma, while the co-patient practice section was filled out by those who regularly provide care or support to asthma patients.

A pilot study was conducted with a small sample from the target population to evaluate the feasibility, effectiveness, and clarity of the questionnaire. This step allowed us to identify and address any issues with question interpretation, data collection procedures, and overall study design, ensuring the reliability and validity of the instrument for the main study. The reliability of the questionnaire was evaluated using Cronbach alpha, with a coefficient of ≥0.6 considered acceptable.^[15]^ Content validity was ensured by Dr Mohamed A. H. Fadul, Senior House Officer at Singa Teaching Hospital and teaching assistant at the University of Khartoum. His feedback helped confirm that the questionnaire effectively captured the key domains of knowledge, attitude, and practice regarding asthma. Additionally, construct validity was assessed by conducting a pilot study, which analyzed the responses for consistency and relevance.

To ensure proper reporting, we followed the STROBE guidelines for cross-sectional studies.^[16]^

### 2.6. Ethics approval and consent to participate

Ethical approval for the study was granted by the Community Medicine Department Review Board at the Faculty of Medicine, University of Khartoum, as part of the rural residency program, where students, under the department’s supervision, conduct community-based research. Administrative approval was also secured from the city mayor. Informed consent was obtained from each participant after a detailed explanation of the study’s purpose and procedures. Participants were informed that they could voluntarily withdraw from the study at any time during the interview.

### 2.7. Data analysis plan

Data entry and statistical analysis were conducted using Statistical Package for the Social Sciences (SPSS®) version 28. Descriptive statistics and frequency tables were employed to summarize the demographic and medical information of the participants. An analysis of missing values indicated that the missing data were random (with EM significant values of 0.247, 0.449, and 0.625 for patients, co-patients, and neither, respectively). Consequently, multiple imputation was applied to handle missing data. The imputation patterns did not show any monotony in the missing value patterns.

The pooled data were used for various analyses. Normality tests for age and scales showed significant deviations from normality (*P* < .01). Therefore, nonparametric tests were used: Mann–Whitney and Kruskal–Wallis tests compared scales with socio-demographic data, while Chi-square and Fisher exact tests assessed associations between the participant type (patient, co-patient, or neither) and responses in the knowledge and attitude sections. Multiple linear regression was employed to explore the relationship between age, gender, marital status, educational level, occupation, and income with the asthma knowledge scale. A *P* value of <.05 was considered statistically significant.

## 3. Results

### 3.1. Socio-demographic characteristics

This study included 148 participants, with a response rate of 67%. All participants completed the socio-demographic, knowledge, and attitude sections. The patient practice section was completed by patients, while the co-patient practice section was filled out by co-patients. Most participants were female (71.14%) with a median age of 36 years. Over half were married (60.54%), and approximately 13% were illiterate. A majority were unemployed (64.19%), and nearly 60% had an income of 50,000 SDGs or more. Nearly half (54.37%) had either been diagnosed with asthma or knew someone with the condition, and about 30% had first- or second-degree relatives with asthma (Table 1).

**Table 1:** The frequency of socio-demographic characteristics across different categories of the participants (asthmatic patients, co-patients, and neither 1 of them) (N = 148).

Characteristic	Patients	Co-patients	Neither	Total (N = 148)
Gender:				
Males Females	1 (0.67%) 9 (6.04%)	9 (6.04%) 26 (17.45%)	33 (22.15%) 71 (47.65%)	43 (28.68%) 106 (71.14%)
Age	*45.00 ± 34.00	*35.00 ± 20.00	*36.00 ± 27.00	*36.00 ± 25.00
Marital status				
Single Married Divorced Widow	2 (1.63%) 4 (2.72%) 1 (0.68%) 1 (0.68%)	10 (6.80%) 23 (15.64%) 0 (0%) 2 (1.63%)	30 (20.41%) 62 (42.18%) 0 (0%) 12 (8.16%)	42 (28.57%) 89 (60.54%) 1 (0.68%) 15 (10.20%)
Educational level				
Illiterate Primary School Middle School High School Higher education	2 (1.35%) 1 (0.68%) 1 (0.68%) 2 (1.35%) 3 (2.03%)	3 (2.03%) 4 (2.70%) 7 (4.73%) 11 (7.43%) 10 (6.76%)	15 (10.14%) 38 (25.68%) 8 (5.41%) 25 (16.89%) 18 (12.16%)	20 (13.51%) 43 (29.05%) 16 (10.81%) 38 (25.68%) 31 (20.95%)
Occupation				
Professional work Skilled work Unemployed	0 (0%) 1 (0.67%) 8 (5.41%)	9 (6.08%) 7 (4.73%) 19 (12.84%)	6 (4.05%) 30 (20.27%) 68 (45.95%)	15 (10.14%) 38 (25.68%) 95 (64.19%)
Income				
<50,000 SDGs 50,000–100,000 SDGs More than 100,000 SDGs	4 (2.70%) 4 (2.70%) 1 (0.68%)	13 (8.78%) 12 (8.11%) 10 (6.76%)	33 (22.30%) 49 (33.11%) 22 (14.86%)	50 (33.78%) 65 (43.92%) 33 (22.30%)
Have you or anyone you know has been diagnosed with asthma:				
Yes No	9 (6.08%) 0 (0%)	28 (18.92%) 7 (4.73%)	30 (20.27%) 74 (50.00%)	67 (45.72%) 81 (54.73%)
Who in your family has been diagnosed with asthma before:				
1st degree relative 2nd degree relative Spouse No one	1 (0.68%) 0 (0%) 0 (0%) 8 (5.41%)	17 (11.49%) 8 (5.41%) 1 (0.68%) 9 (6.08%)	6 (4.05%) 15 (10.14%) 0 (0%) 83 (56.08%)	24 (16.22%) 23 (15.54%) 1 (0.68%) 100 (67.57%)

SDG = Sudanese pounds.

* Median ± interquartile range.

### 3.2. Knowledge and attitude about asthma

Over half of the participants (60%) believed that a vaporizer is an effective treatment for asthma, 85.81% thought it is good to have asymptomatic asthma visits, 79.05% saw asthma as a common cause of school absences, 69.59% believed asthma runs in families, and 98.65% agreed that treatment should be sought in a hospital. Additionally, 60.14% reported that asthma should not limit exercise, 65.54% felt asthma is not an emotional illness, and 78.38% believed asthma does not resolve just because an attack stops. However, 41% thought asthma could be cured, and 43% believed it always begins in childhood.

Regarding the signs and triggers of asthma, more than (90%) of the participants reported the following as asthma signs: shortness of breath (97.30%), chest tightness (95.95%), nocturnal cough (90.54%), and wheezing with exercise (90.54%). Almost half of the participants thought that severe headaches could be a sign of asthma (52.03%). More than (90%) reported animal contact (91.22%) and the harvest season (95.95%) as triggers for asthma. Almost half reported cockroaches and poor diet as potential asthma triggers (47.30% and 56.76%, respectively). Surprisingly, almost (81.76%) of the participants stated that mosquitoes cannot trigger asthma.

Attitudes toward asthma showed that more than 70% felt asthmatics should avoid sports (75%), be educated on managing acute attacks (96.62%), have school-based educational programs (97.30%), and recognize asthma’s impact on work (78.38%). Knowledge and attitude responses were generally consistent across participant groups (patients, co-patients, and neither), with significant differences found only in treatment-seeking behavior (*P* = .032) and the belief that cockroaches can trigger asthma (*P* = .010), with co-patients giving more correct answers (Table 2).

**Table 2:** The frequency of asthma knowledge and attitude characteristics across different categories of the participants (asthmatic patients, co-patients, and neither 1 of them) (N = 148).

Variables	Patient	Co-patient	Neither	Total	*P* value
Knowledge about asthma					
Asthma can’t be cured:					.418
False True	3 (2.03%) 6 (4.05%)	11 (7.43%) 24 (16.22%)	47 (31.76%) 57 (38.51%)	61 (41.22%) 87 (58.78%)	
Vaporizer is a good asthma treatment:					.832
False True	1 (0.68%) 8 (5.41%)	6 (4.05%) 29 (19.59%)	14 (9.46%) 90 (60.81%)	21 (14.19%) 127 (85.81%)	
Asthma shouldn’t limit exercise:					.919
True False	4 (2.70%) 5 (3.38%)	13 (8.78%) 22 (14.86%)	42 (28.38%) 62 (41.89%)	59 (39.86%) 89 (60.14%)	
There is a need for asymptomatic asthma visits:					.531
False True	3 (2.03%) 6 (4.05%)	6 (4.05%) 29 (19.59%)	22 (14.86%) 82 (55.41%)	31 (20.95%) 117 (79.05%)	
Asthma is a common reason for school absence:					.098
False True	0 (0%) 9 (6.08%)	12 (8.11%) 23 (15.54%)	33 (22.30%) 71 (47.97%)	45 (30.41%) 103 (69.59%)	
Does asthma run in families?					.361
True False	8 (5.41%) 1 (0.68%)	23 (15.54%) 12 (8.11%)	82 (55.41%) 22 (14.86%)	113 (77.03%) 35 (23.65%)	
Asthma is mainly an emotional illness:					.521
True False	2 (1.35%) 7 (4.73%)	9 (6.08%) 26 (17.57%)	40 (27.03%) 64 (43.24%)	51 (34.46%) 97 (65.54%)	
Asthma resolves if the asthma attack stops:					.592
True False	2 (1.35%) 7 (4.73%)	9 (6.08%) 26 (17.57%)	21 (14.19%) 83 (56.08%)	32 (21.62%) 116 (78.38%)	
Asthma onset is always in childhood:					.505
True False	3 (2.03%) 6 (4.05%)	13 (8.78%) 22 (14.86%)	49 (33.11%) 55 (37.16%)	65 (43.92%) 83 (56.08%)	
Where to go for treatment?					.032*
Local helper Hospital	1 (0.68%) 8 (5.41%)	1 (0.68%) 34 (22.97%)	0 (0%) 104 (70.27%)	2 (1.35%) 146 (98.65%)	
Is a shortness of breath a sign or symptom of asthma?					.668
No Yes	0 (0%) 9 (6.08%)	0 (0%) 35 (23.65%)	4 (2.70%) 100 (67.57%)	4 (2.70%) 144 (97.30%)	
Is chest tightness a sign or symptom of asthma?					.283
No Yes	0 (0%) 9 (6.08%)	0 (0%) 35 (23.65%)	6 (4.05%) 98 (66.22%)	6 (4.23%) 142 (95.95%)	
Are severe headaches signs or symptoms of asthma?					.118
Yes No	3 (2.03%) 6 (4.05%)	23 (15.54%) 12 (8.11%)	51 (34.46%) 53 (35.81%)	77 (52.03%) 71 (47.97%)	
Is a nocturnal cough sign or symptoms of asthma?					.436
No Yes	0 (0%) 9 (6.08%)	2 (1.35%) 33 (22.30%)	12 (8.11%) 92 (62.16%)	14 (9.46%) 134 (90.54%)	
Is wheezing with exercise a sign or symptom of asthma?					.534
No Yes	0 (0%) 9 (6.08%)	2 (1.35%) 33 (22.30%)	12 (8.11%) 92 (62.16%)	14 (9.46%) 134 (90.54%)	
Can animal contact (including household animals) trigger asthma?					.248
No Yes	0 (0%) 9 (6.08%)	1 (0.68%) 34 (22.97%)	12 (8.11%) 92 (62.16%)	13 (8.78%) 135 (91.22%)	
Can mosquito bites trigger asthma?					.176
No Yes	7 (4.73%) 2 (1.35%)	25 (16.89%) 10 (6.76%)	89 (60.14%) 15 (10.14%)	121 (81.76%) 27 (18.24%)	
Can dampness trigger asthma?					.160
No Yes	1 (0.68%) 8 (5.41%)	9 (6.08%) 26 (17.57%)	41 (27.70%) 63 (42.57%)	51 (34.46%) 97 (65.54%)	
Can cockroaches trigger asthma?					.010*
No Yes	7 (4.73%) 2 (1.35%)	11 (7.43%) 24 (16.22%)	60 (40.54%) 44 (29.73%)	78 (52.70%) 70 (47.30%)	
Can a poor diet trigger asthma?					.242
No Yes	5 (3.38%) 4 (2.70%)	11 (7.43%) 24 (16.22%)	48 (32.43%) 56 (37.84%)	64 (43.24%) 84 (56.76%)	
Can harvest season trigger asthma?					.433
No Yes	1 (0.68%) 8 (5.41%)	1 (0.68%) 34 (22.97%)	(2.70%) 100 (67.57%)	6 (4.05%) 142 (95.95%)	
Attitude regarding asthma:					
Asthmatic patients should avoid sports activities and physical education classes.					.893
Agree Disagree	7 (4.73%) 2 (1.35%)	25 (16.89%) 10 (6.76%)	79 (53.38%) 25 (16.89%)	111 (75.00%) 37 (25.00%)	
Asthmatic patients should avoid certain foods					.636
Agree Disagree	3 (2.03%) 6 (4.05%)	17 (11.49%) 18 (12.16%)	52 (35.14%) 52 (35.14%)	72 (48.65%) 76 (51.35%)	
Asthmatic patients should be educated on how to manage an acute asthmatic attack:*					.168
Disagree Agree	0 (0%) 9 (6.08%)	3 (2.03%) 32 (21.62%)	2 (1.35%) 102 (68.92%)	5 (3.39%) 143 (96.62%)	
The frequent use of antibiotics helps in diminishing the complications of asthma:					.297
Agree Disagree	7 (4.73%) 2 (1.35%)	18 (12.16%) 17 (11.49%)	66 (44.59%) 38 (25.68%)	91 (61.49%) 57 (38.51%)	
There is a need to create educative programmes for schools:					.346
Disagree Agree	1 (0.68%) 8 (5.41%)	1 (0.68%) 34 (22.97%)	2 (1.35%) 102 (68.92%)	4 (2.70%) 144 (97.30%)	
Do you think asthma affects the work aspect of life?					.885
Yes No	8 (5.41%) 1 (0.67%)	27 (18.24%) 8 (5.41%)	81 (54.73%) 23 (15.54%)	116 (78.38%) 32 (21.62%)	
Do you think asthma affects the social aspect of life?					.919
Yes No	4 (2.70%) 5 (3.38%)	14 (9.46%) 21 (14.19%)	46 (31.08%) 58 (39.19%)	64 (43.24%) 84 (56.76%)	
Do you think asthma affects the marital status aspect of life?					1.00
Yes No	3 (2.03%) 6 (4.05%)	11 (7.43%) 24 (16.22%)	35 (23.65%) 69 (46.62%)	49 (33.10%) 99 (66.89%)	

* *P* value <.05 (significant).

### 3.3. Practice regarding asthma

Nine individuals filled out the practice section in the questionnaire. Five of them (55.55%) were using the prescribed inhaler and also committing to regular follow-up (55.55%). All of them reported going to the hospital for acute attacks and consenting to receive oxygen therapy when needed.

Regarding the co-patients point of view, 35 co-patients filled out this section. Overall, (80%) of them stated that they help the individual use the inhaler, approve of oxygen therapy for the individual when needed (97.14%), assist with treatment and follow-up (88.57% and 82.86%, respectively), and help the individual go to the hospital during an acute asthma attack (97.14%) (Table 3).

**Table 3:** The frequency of asthma practice characteristics across asthmatic patients and co-patients (N = 9 for patients and 35 for co-patients).

Practice characteristics	Frequency (%)
Patients perspective:	
Do you use the prescribed inhaler?	
No Yes	4 (44.44%) 5 (55.55%)
When you require oxygen therapy do you consent to receiving it?	
No Yes	0 (0%) 9 (100%)
When you require daily therapy do you commit to the treatment?	
No Yes	2 (22.22%) 7 (77.78%)
During an acute asthma attack do you go to the hospital?	
No Yes	0 (0%) 9 (100%)
Do you commit to regular follow-up visits to the doctor?	
No Yes	4 (44.44%) 5 (55.55%)
Co-patient perspective:	
Do you help/tell the patient to use the prescribed inhaler?	
No	7 (20.00%)
Yes	28 (80.00%)
When the patient requires oxygen therapy, do you approve of them receiving it?	
No Yes	1 (2.86%) 34 (97.14%)
When they require daily therapy, do you help them commit to the treatment?	
No Yes	4 (11.43%) 31 (88.57%)
During an acute asthma attack do you help the patient go to the hospital?	
No Yes	0 (0%) 35 (100%)
Do you help them commit to regular follow-up visits to the doctor?	
No Yes	6 (17.14%) 29 (82.86%)

### 3.4. Factors affecting the level of knowledge, attitude, and practice about asthma

Knowledge, practice, and attitude scales were assessed with higher scores reflecting better knowledge, a positive attitude, and good practice. Significant associations with the knowledge scale were found for gender, marital status, occupation, and income (*P* values = .040, .003, .000, and .006, respectively). The highest median knowledge scores were reported among divorced individuals, those with higher education, professional workers, and people with incomes below 50,000 SDGs (medians = 17, 16, 17, and 16, respectively). No significant difference in medians was observed between genders.

Attitude scale differences were significant across marital status, educational level, occupation, and the presence of a family member with asthma (*P* values = .001, .000, .003, and .016, respectively). The highest median attitude scores were among divorced individuals, those with high school or higher education, professionals, and those with a first-degree relative with asthma (medians = 6, 5, 5, and 5, respectively).

The patient practice scale showed significant differences related to marital status (*P* value = .026) and income (*P* value = .006). For the co-patient practice scale, significant factors included occupation, having asthma or knowing someone with asthma, and the identity of the family member with asthma (*P* values = .026, .003, and .000, respectively). The highest median patient practice scores were observed among singles and those with incomes below 50,000 SDGs (median = 4.5 for both). No significant differences were found in co-patient practice scale medians among different groups (Table 4).

**Table 4:** Univariate analysis of socio-demographic characteristics across the asthma knowledge scale, attitude scale, patient practice scale and co-patient practice scale (N = 148).

Characteristic	Knowledge scale		Attitude scale		†Patient practice		‡Co-patient practice	
	*Median ± IQR	*P* value	Median ± IQR	*P* value	Median ± IQR	*P* value	Median ± IQR	*P* value
Gender: Males Females	15.00 ± 0.37 15.00 ± 0.00	.040	4.00 ± 0.00 4.00 ± 0.25	.70	4.00 ± 0.00 4.00 ± 0.00	1.00	5.00 ± 0.00 5.00 ± 0.00	.889
Age	§		§		§		§	
Marital status: Single Married Divorced Widow	15.00 ± 0.00 15.00 ± 0.00 17.00 ± 0.00 15.00 ± 0.00	.003	5.00 ± 0.00 4.00 ± 1.00 6.00 ± 0.00 5.00 ± 0.00	.001	4.50 ± 0.00 4.00 ± 0.00 3.00 ± 0.00 5.00 ± 0.00	.026	5.00 ± 0.00 5.00 ± 0.00 § 5.00 ± 0.00	.787
Educational level: Illiterate Primary School Middle School High School Higher education	15.00 ± 0.33 15.00 ± 0.67 15.50 ± 0.00 14.00 ± 0.67 16.00 ± 0.00	.061	3.41 ± 0.00 4.00 ± 0.00 4.00 ± 0.00 5.00 ± 0.00 5.00 ± 0.00	.000	3.00 ± 0.00 3.00 ± 0.00 4.00 ± 0.00 4.50 ± 0.00 4.00 ± 0.00	.410	5.00 ± 0.00 5.00 ± 0.00 5.00 ± 0.00 5.00 ± 0.00 5.00 ± 0.00	.014
Occupation: Professional work Skilled work Unemployed	17.00 ± 0.00 15.17 ± 0.00 15.00 ± 0.00	.000	5.00 ± 0.00 4.00 ± 0.00 4.24 ± 0.00	.003	§ 4.00 ± 0.00 4.00 ± 0.00	1.00	5.00 ± 0.00 5.00 ± 0.00 5.00 ± 0.00	.026
Income: <50,000 SDGs 50,000–100,000 SDGs More than 100,000 SDGs	16.00 ± 0.00 15.00 ± 0.63 15.00 ± 1.00	.006	4.00 ± 0.00 5.00 ± 0.00 4.00 ± 0.00	.729	4.50 ± 0.00 4.00 ± 0.00 2.00 ± 0.00	.006	5.00 ± 0.00 5.00 ± 0.00 5.00 ± 0.00	.736
Have you or anyone you know been diagnosed with asthma: Yes No	15.00 ± 0.00 15.00 ± 0.00	.807	4.36 ± 0.00 4.00 ± 0.00	.727	4.00 ± 0.00 §	§	5.00 ± 0.00 5.00 ± 0.00	.003
Who in your family has been diagnosed with asthma before: 1st degree relative 2nd degree relative Spouse No one	15.00 ± 0.33 15.00 ± 0.00 17.00 ± 0.00 15.00 ± 0.00	.958	5.00 ± 0.00 4.00 ± 1.00 3.00 ± 0.00 4.00 ± 0.00	.016	4.00 ± 0.00 § § 4.00 ± 0.00	1.00	5.00 ± 0.00 5.00 ± 0.00 5.00 ± 0.00 5.00 ± 0.00	.000

SDG = Sudanese pounds.

* Interquartile range.

† Only patients filled this section (N = 9).

‡ Only co-patients filled this section (N = 35).

§ Not applicable.

### 3.5. Regression model for knowledge scale

Multiple linear regression was performed for numerous socio-demographic factors regarding the knowledge scale as the dependent variable. The model was significant (*P* value = .00) and the adjusted *R*^2^ was 0.166. In the model, the following were found to be significantly affecting the scale: age, gender, educational level, and occupation (*P* value = .008, .031, .035 and .003 respectively). All of these factors had a direct relation with the scale (positive B value) except occupation which had an inverse relationship (negative B value) (Table 5).

**Table 5:** Multiple regression analysis of socio-demographic factors regarding the asthma knowledge scale (N = 148).

Variables	Unstandardized coefficients		t	*P* value	95.0% confidence interval for B		Fraction missing info.	Relative increase variance	Relative efficiency
	B	Std. error			Lower bound	Upper bound			
Constant	13.531	1.231	10.996	.000	11.099	15.964	.180	.203	.965
Gender	.882	.406	2.176	.031	.083	1.682	.140	.153	.973
Age	.032	.012	2.647	.008	.008	.056	.088	.093	.983
Marital status	.139	.239	.580	.562	-.331	.609	.060	.062	.988
Educational level	.286	.135	2.122	.035	.020	.552	.164	.182	.968
Occupation	-.755	.252	-2.998	.003	-1.248	-.261	.007	.007	.999
Income	-.172	.231	-.746	.457	-.629	.284	.168	.187	.968

## 4. Discussion

Assessing population knowledge, attitudes, and practices is essential for improving disease management, as better understanding can lead to reduced disease severity and morbidity.^[17]^ This study aimed to evaluate the knowledge, attitudes, and practices related to asthma among the adults in Shendi locality, Northern Sudan.

The research focused on adult residents of Kabushiya, with a participant pool predominantly consisting of women. This gender imbalance likely reflects community characteristics and research constraints, as most men were at work during the daytime survey.

The analysis revealed that several socio-demographic factors, including gender, marital status, occupation, and income significantly influenced asthma knowledge. Those who were divorced, had higher education, worked in professional occupations, or had lower incomes tended to have better knowledge. Particularly, individuals in these categories showed stronger understanding, though this could be partly due to the small sample size for certain groups.

In our study, divorced individuals exhibited higher asthma knowledge compared to their married counterparts. A plausible explanation could be that divorced individuals, particularly in regions like Sudan, often assume more responsibilities, including managing their own and their children’s health, which drives them to seek more health-related information. A similar trend was observed in a population-based cohort study, where separated participants showed greater engagement in health-related behaviors and better health outcomes,^[18]^ possibly due to the absence of spousal support.

The association between income and asthma knowledge in our study revealed that participants with lower incomes demonstrated better asthma-related knowledge compared to wealthier individuals. This may be attributed to higher asthma prevalence in low-income groups,^[19,20]^ which leads to more frequent interactions with healthcare providers and subsequent education on managing the condition. As a result, these individuals may acquire more comprehensive knowledge about asthma.

In contrast, research from rural South India suggests that a higher socioeconomic status contributes to better health knowledge and healthier families,^[21]^ highlighting a different trend.

In this study, participants with higher education levels showed greater asthma knowledge, likely due to enhanced access to reliable health information and resources, such as healthcare professionals, educational materials, and technology. Education improves health literacy, empowering individuals to understand and manage medical conditions more effectively. This aligns with other research, such as a study in Chicago, where lower educational attainment was linked to reduced asthma awareness, likely due to limited access to health education and resources.^[17]^ The regression model highlighted age, gender, educational attainment, and occupation as key factors influencing knowledge, with males generally exhibiting higher knowledge levels, reflecting ongoing educational disparities in rural areas.

Older and better-educated participants were more knowledgeable, likely due to accumulated life experience and improved access to education. This is consistent with findings from a study in China, which showed that parental education positively influenced knowledge, attitudes, and practices regarding asthma.^[22]^ Similarly, research in Khartoum asthma clinics found a strong correlation between mothers’ educational attainment and their knowledge levels.^[13]^ Conversely, a study in New York City revealed that younger, better-educated individuals were more knowledgeable about asthma.^[23]^ In this research, age and education were positively linked to knowledge, while occupation had an inverse relationship.

Most participants identified symptoms such as shortness of breath, wheezing, nocturnal cough, and chest tightness as indicative of asthma, aligning with findings from rural India.^[21]^ Additionally, many participants recognized environmental factors, such as the harvest season and animal contact, as asthma triggers. However, misconceptions persist; for instance, a notable portion of participants in this study mistakenly identified headaches as a symptom of asthma, a misconception less common in the rural India study.^[21]^ Furthermore, while the majority correctly understood that asthma is not an emotional illness, a significant portion held the incorrect belief that asthma could be cured. This misconception likely stems from a lack of health education programs and the stigma associated with asthma in the region, similar to findings from Pakistan.^[24]^

The analysis of attitudes towards asthma revealed notable differences based on marital status, occupation, educational attainment, and whether a family member had asthma. Those who were divorced, had completed high school or further education, worked in professional roles, or had a close family member with asthma displayed more positive attitudes.

Almost all participants believed that asthmatic patients should seek treatment at the hospital, contrasting with a study in rural Pakistan where a significant portion preferred alternative medicine or only sought hospital care during acute attacks.^[24]^ Similarly, nearly all participants supported the idea of including asthma education in schools. However, a large majority felt that people with asthma should avoid sporting activities.

Differences in treatment-seeking behavior and the belief that cockroaches could trigger asthma were observed, with those who had direct experience with asthma, either as patients or through family members, showing higher levels of correct understanding. This finding aligns with a study in Chicago, which also found that individuals with personal or familial experience had better knowledge.^[17]^

The participants demonstrated relatively good practices regarding asthma treatment and where to seek it, though the reliability of this data may be influenced by the limited number of relevant responses, as the practice section was only applicable to those who were asthmatic or had cared for someone with asthma. Out of the total participants, a small group matched this description.

Significant differences in asthma practices were observed among patients based on their income and marital status. Those with lower incomes and single individuals tended to show better adherence to asthma management practices. This trend could be influenced by the smaller sample size in these groups. Additionally, it is possible that individuals with lower incomes are more diligent in managing their asthma to prevent the financial burden of costly complications.

For those caring for asthmatics, the practices were significantly influenced by whether they were close to someone with asthma, had the condition themselves, or were in certain occupations. However, there were no significant differences between the different co-patient groups.

About half of the asthmatic patients reported using prescribed inhalers, and a majority of co-patients stated they assisted in inhaler use, contrasting sharply with a study in rural Pakistan where a much smaller percentage used inhalers.^[24]^ The lower usage in that study was attributed to negative perceptions, such as the belief that asthma medications cause addiction or social stigma associated with inhaler use.^[21,25]^

Despite the generally good practices observed in our study, several barriers may still impede patients from accessing appropriate asthma management. One of the key barriers is asthma-related stigma,^[26]^ which can deter patients from seeking care. This stigma often stems from misconceptions about asthma being a sign of weakness or an emotional condition, making individuals reluctant to disclose their condition or seek help. Additionally, limited healthcare availability,^[27]^ especially in rural areas, restricts access to specialized care and regular follow-up services, which are crucial for managing asthma effectively. Furthermore, economic constraints significantly affect access to asthma treatment.^[28]^ People from lower-income backgrounds may face challenges affording medications or prioritizing follow-up visits, often placing financial survival over health needs. These barriers, taken together, suggest that even good knowledge and practices around asthma do not always lead to optimal management outcomes.

### 4.1. Strengths and limitations

A major strength of our research is its focus on the community, which provides valuable insights into the knowledge, attitude, and practice of asthma within Shendi locality. However, as a cross-sectional study, it cannot determine cause-and-effect relationships or track changes in behavior over time. Additionally, since the study was conducted during the day, not all adults were available to participate, which may have introduced selection bias. Furthermore, the findings may have limited generalizability beyond the study population due to the specific geographic and demographic characteristics of Shendi locality. Another limitation of this study is the reliance on self-reported data, which may introduce bias, particularly with regard to overestimating knowledge or adherence to asthma management practices. This effect may be more pronounced in a face-to-face interview setting, despite efforts to minimize social desirability bias by ensuring confidentiality and training interviewers to maintain neutrality.

To overcome these limitations, future studies should use a longitudinal approach and consider sampling strategies that allow for broader generalization of results.

## 5. Conclusion

Several socio-demographic and socioeconomic factors, particularly educational level and occupation, were associated with asthma knowledge, attitudes, and practices. Most respondents lacked sufficient knowledge and had misconceptions about asthma and its treatments. This gap in understanding may result in individuals in Kabushiya village not seeking timely medical help or following effective treatment plans, potentially leading to poorly controlled asthma, more frequent severe attacks, and a lower quality of life.

However, aside from the use of inhalers and routine follow-ups, participants generally showed appropriate practices concerning therapy and where to obtain it. Community education plays a crucial role in improving disease outcomes. Physicians are key in not only providing accurate information about asthma but also in changing community behavior and practices related to disease management. To improve knowledge, attitudes, and practices, future efforts should focus on enhancing health education in schools and increasing public awareness through campaigns.

## Acknowledgments

We would like to express our gratitude to the Department of Community Medicine at the Faculty of Medicine, University of Khartoum, for their invaluable support.

## Author contributions

**Conceptualization:** Sara Elawad, Sabaa I. Saad-Omer, Sahar A. A. Ahmed, Sara B. Bashir, Selma A. H. Abdelmutaleb, Sarah O. Mohamed, Suad B. Babiker, Sara MS. Osman, Mohamed A. H. Fadul, Esameldeen O. M. Abdalkareem.

**Data curation:** Mohamed H. Elbadawi.

**Formal analysis:** Mohamed H. Elbadawi.

**Investigation:** Sara Elawad, Sabaa I. Saad-Omer, Sahar A. A. Ahmed, Sara B. Bashir, Selma A. H. Abdelmutaleb, Sarah O. Mohamed, Suad B. Babiker, Sara MS. Osman, Mohamed A. H. Fadul, Esameldeen O. M. Abdalkareem, Ali Galal.

**Methodology:** Sara Elawad, Sabaa I. Saad-Omer, Shaima Omer Mohamed Elawad, Sahar A. A. Ahmed, Sara B. Bashir, Selma A. H. Abdelmutaleb, Sarah O. Mohamed, Suad B. Babiker, Sara MS. Osman, Mohamed A. H. Fadul, Esameldeen O. M. Abdalkareem, Ali Galal.

**Supervision:** Mohamed A. H. Fadul.

**Validation:** Mohamed H. Elbadawi.

**Writing – original draft:** Sara Elawad, Sahar A. A. Ahmed, Sara B. Bashir, Selma A. H. Abdelmutaleb, Sarah O. Mohamed, Mohamed A. H. Fadul, Esameldeen O. M. Abdalkareem.

**Writing – review & editing:** Sabaa I. Saad-Omer, Shaima Omer Mohamed Elawad, Ahmed Balla M. Ahmed, Ali Galal.

## References

[1] Hashmi MF Cataletto ME. Asthma. In: StatPearls. Treasure Island (FL): StatPearls Publishing; 2024 [May 3, 2024].

[2] Brigham EP West NE. Diagnosis of asthma: diagnostic testing. Int Forum Allergy Rhinol. 2015;5(Suppl 1):S27–30. 26335833 10.1002/alr.21597

[3] Schleich F Bougard N Moermans C Sabbe M Louis R. Cytokine-targeted therapies for asthma and COPD. Eur Respir Rev. 2023;32:220193. 37076177 10.1183/16000617.0193-2022 PMC10113955

[4] Reports - Global Initiative for Asthma - GINA. Global Strategy for Asthma Management and Prevention. Published 2016. https://ginasthma.org/reports/. Accessed October 3, 2024.

[5] Adeloye D Chan KY Rudan I Campbell H. An estimate of asthma prevalence in Africa: a systematic analysis. Croat Med J. 2013;54:519–31. 24382846 10.3325/cmj.2013.54.519 PMC3893990

[6] Ait-Khaled N Odhiambo J Pearce N. Prevalence of symptoms of asthma, rhinitis and eczema in 13- to 14-year-old children in Africa: the International Study of Asthma and Allergies in Childhood Phase III. Allergy. 2007;62:247–58. 17298341 10.1111/j.1398-9995.2007.01325.x

[7] Kabra SK Lodha R. Long-term management of asthma. Indian J Pediatr. 2003;70:63–72. 12619955 10.1007/BF02722747

[8] Sidhu GS Garg K Chopra V. Stigma and self-esteem in patients of bronchial asthma [published online ahead of print October 9, 2023]. Monaldi Arch Chest Dis. doi: 10.4081/monaldi.2023.2711. 10.4081/monaldi.2023.2711 37817740

[9] Ilmarinen P Stridsman C Bashir M. Level of education and asthma control in adult-onset asthma. J Asthma. 2022;59:840–9. 33497270 10.1080/02770903.2021.1871742

[10] The Global Asthma Report. Global Asthma Network (GAN). Int J Tuberc Lung Dis. 2022;26:S1–S102. 10.5588/ijtld.22.1010 36303302

[11] Osman R Ahmed K ElSony A. Factors associated with uncontrolled asthma among Sudanese adult patients. J Pan Afr Thorac Soc. 2021;2:85–93.

[12] Noureddin AA Shaaban KM Mohamed SOO Abdalla AA Mahmoud AAA Salman MST. The knowledge attitude and practice (KAP) of mothers of asthmatic children toward asthma in Khartoum asthma clinics. Sci Rep. 2019;9:12120. 31431663 10.1038/s41598-019-48622-2 PMC6702345

[13] Naing L Winn T Rusli BN. Practical issues in calculating the sample size for prevalence studies. Archives of Orofacial Sciences; 2006;1:9–14.

[14] The DHS Program. Calculate sample size using survey sampling online tools: one sample - the DHS program blog. The DHS Program Blog. 2023. https://blog.dhsprogram.com/calculate-sample-size-using-survey-sampling-online-tools-one-sample/. Accessed October 3, 2024.

[15] Ursachi G Horodnic IA Zait A. How reliable are measurement scales? External factors with indirect influence on reliability estimators. Procedia Econ Financ. 2015;20:679–86.

[16] Von Elm E Altman DG Egger M Pocock SJ Gøtzsche PC Vandenbroucke JP. STROBE initiative. The strengthening the reporting of observational studies in epidemiology (STROBE) statement: guidelines for reporting observational studies. Lancet. 2007;370:1453–7. 18064739 10.1016/S0140-6736(07)61602-X

[17] Malone AM Gupta RS Lyttle CS Weiss KB. Characterizing community-based asthma knowledge in Chicago and its high-risk neighbourhoods. J Asthma. 2008;45:313–8. 18446596 10.1080/02770900801911202

[18] Wang L Yi Z. Marital status and all-cause mortality rate in older adults: a population-based prospective cohort study. BMC Geriatr. 2023;23:214. 37016371 10.1186/s12877-023-03880-8 PMC10074686

[19] Schyllert C Lindberg A Hedman L. Low socioeconomic status relates to asthma and wheeze, especially in women. ERJ Open Res. 2020;6:00258–2019. 32963998 10.1183/23120541.00258-2019 PMC7487352

[20] Kant S. Socio-economic dynamics of asthma. Indian J Med Res. 2013;138:446–8. 24434252 PMC3868058

[21] Daniel J Inbaraj LR Jenkins S Ramamurthy PH Isaac R. A community-based cross-sectional study on knowledge, attitude, and perceptions about asthma among healthy adults in rural South India. J Family Med Prim Care. 2021;10:1956–62. 34195131 10.4103/jfmpc.jfmpc_2152_20 PMC8208207

[22] Zhao J Shen K Xiang L. The knowledge, attitudes and practices of parents of children with asthma in 29 cities of China: a multi-centre study. BMC Pediatr. 2013;13:2–7. 23379859 10.1186/1471-2431-13-20 PMC3577449

[23] Mancuso CA Rincon M. Impact of health literacy on longitudinal asthma outcomes. J Gen Intern Med. 2006;21:813–7. 16881939 10.1111/j.1525-1497.2006.00528.x PMC1831574

[24] Zaman M Ashraf S Javaid M. Reliability of diagnosis and asthma knowledge, attitudes and perception (KAP) in rural population of NWFP, Pakistan. Pakistan Journal of Chest Medicine. 2006;12.

[25] Gupta VK Bahia JS Maheshwari A Arora S Gupta V Nohria S. To study the attitudes, beliefs and perceptions regarding the use of inhalers among patients of obstructive pulmonary diseases and in the general population in Punjab. J Clin Diagn Res. 2011;5:434–9.

[26] Ahmad S Ismail NE. Stigma in the lives of asthma patients: a review from the literature. Int J Pharm Pharm Sci. 2015;7:40–6. https://journals.innovareacademics.in/index.php/ijpps/article/view/6100/7107. Accessed October 4, 2024.

[27] Codispoti CD Greenhawt M Oppenheimer J. The role of access and cost-effectiveness in managing asthma: a systematic review. J Allergy Clin Immunol Pract. 2022;10:2109–16. 35525532 10.1016/j.jaip.2022.04.025 PMC9353043

[28] Zeitouni M Al-Moamary M Coussa M. Challenges and recommendations for the management of asthma in the Middle East and Africa. Ann Thorac Med. 2022;17:71. 35651897 10.4103/atm.atm_469_21 PMC9150662

